# Characterizing the Solar Activity Using the Visibility Graph Method

**DOI:** 10.3390/e25020342

**Published:** 2023-02-13

**Authors:** Tomás Zurita-Valencia, Víctor Muñoz

**Affiliations:** Departamento de Física, Facultad de Ciencias, Universidad de Chile, Las Palmeras 3425, Ñuñoa, Santiago 7800003, Chile

**Keywords:** solar activity, magnetic field, sunspots, time series analysis, visibility graph, complex networks, complexity, centrality, decay exponent

## Abstract

In this paper, the Sun and its behavior are studied by means of complex networks. The complex network was built using the Visibility Graph algorithm. This method maps time series into graphs in which every element of the time series is considered as a node and a visibility criterion is defined in order to connect them. Using this method, we construct complex networks for magnetic field and sunspots time series encompassing four solar cycles, and various measures such as degree, clustering coefficient, mean path length, betweenness centrality, eigenvector centrality and decay exponents were calculated. In order to study the system in several time scales, we perform both a global, where the network contains information on the four solar cycles, and a local analysis, involving moving windows. Some metrics correlate with solar activity, while others do not. Interestingly, those metric which seem to respond to varying levels of solar activity in the global analysis, also do in the moving windows analysis. Our results suggest that complex networks can provide a useful way to follow solar activity, and reveal new features on solar cycles.

## 1. Introduction

Various measures of complexity can provide relevant ways to study the dynamics of magnetized plasma and, in particular, complex networks have been largely used to study a vast number of physical systems [1,2], as their graph representation has been found to be helpful to characterize and model their phenomenology. Complementing these studies, mathematical tools from statistical physics have also proven to be particularly suitable for studying and understanding complex networks [3].

These works show that the subjacent phenomenology in various systems can be inferred from its complex behavior, thus suggesting the great potential of complex networks to tackle problems in a variety of fields, such as economy [4,5,6,7,8], biology [9,10], or in the study of geophysical problems such as earthquakes, magnetic storms or atmospheric flows [11,12,13,14,15], which prove the versatility of the method and its robustness.

The Sun is a particularly interesting system to study from the point of view of complexity. The interaction of particles and magnetic fields in the Sun’s plasma, leads to a nonlinear dynamics which, in turns, leads to varying levels of solar activity, as manifested in the evolution of sunspots on the Sun’s photosphere, velocity and turbulence levels of the solar wind, events such as solar flare or coronal mass ejections, etc. Since the Sun is our closest star, it is essential to understand its behavior and the impact of solar activity on our planet, especially the impact of its magnetic activity and its effects on the Earth through the Earth’s magnetic field and solar wind coupling [16,17], which may lead to intense geomagnetic storms that may affect human communications and spacecrafts in periods of high solar activity [18].

Various complexity analyses have been carried out to study this rich behavior. For instance, fractal and multifractal features have been identified in the Sun’s photosphere, which have been shown to correlate with the evolution of solar activity [19], and have been proposed to be related to the emergence of solar flares [20,21,22].

Other works have focused on the chaotic and persistent features of the sunspots time series [23,24]. Also, self-organized critical models have been proposed to represent the Sun’s flare activity [25] and its power-law statistics [26].

In this work, we intend to follow a different approach, based on complex networks. Various recent works have carried out complex networks analysis to study the Sun’s activity, focusing on its major features: sunspots. For instance, in Ref. [27], the spatiotemporal patterns of sunspots are mapped into a complex network, showing that some topological measures of the network correlate with the solar cycle, while others anticorrelate, or remain essentially invariant. This is consistent with the fact that different measures inform about different features of the network topology, so that some measures vary in response to the changes in sunspots number and location, whereas others point at complex properties which remain invariant along the solar cycle.

The previous work, maps the spatiotemporal evolution of the sunspots distribution into a complex network. Nodes represent their location, and links represent their time sequence. However, various works have shown that valuable information about complex systems can be extracted by focusing on the time domain, by mapping time series into complex networks.

This was introduced by Lacasa et al. [28], and thanks to this and other works, it has been established that the resulting complex network has topological properties that reflect properties of the original time series [29,30]. Thus, the Visibility Graph method (see details in Section 2.3.1) becomes an interesting tool, allowing, through the study of complex networks, to infer properties of the underlying dynamics. In the context of space physics, Suyal et al. [31] applied to analyze the solar wind, a turbulent plasma whose origin is the upper atmosphere of the Sun and which leads to dynamic phenomena throughout the heliosphere on various temporal and spatial scales. In the following years, several authors have further explored the use of VG to various issues related to space and astrophysical physics, such as the analysis of reversibility in the turbulent states of solar wind simulations [32], the analysis of high-energy emission mechanisms of blazars [33], characterization of sunspot time series [34], statistical studies of solar flares [35], discrimination between types of variable pulsating stars [36], among others.

In particular, the work of Zou et al. [34] is interesting, since the VG analysis provides a complexity perspective to the analysis of the number of sunspots, which has been the traditional indicator of solar activity for centuries. There, the authors perform a global analysis, constructing the VG from the complete time series of the number of sunspots, from the mid-nineteenth century to the first decade of the twentieth century.

Nonetheless, since the solar magnetic activity is not constant, which manifests itself, e.g., in 11-year cycles [37], it is also relevant to study the complex properties of the sunspot configuration as a function of time. For example, it has been shown how the fractal dimension of the solar photosphere correlates with solar activity [19], and more recently, it has been studied how complex networks constructed from the spatio-temporal configuration of sunspots, also present various metrics that correlate or anti-correlate with the solar cycle [27].

Considering these results, we propose to carry out a VG study of the sunspot time series, using moving time windows to establish whether the complexity of this time series and its evolution provide information about variations in solar activity, complementing similar results based on fractal dimensions and complex networks [19,27].

We also notice that both works just mentioned are based on image analysis of solar magnetograms. However, these images are actually a representation of the magnitude of the solar magnetic field, so, as a first approach to consider the physical information contained in the magnetic field itself, we will analyze, in this work, the time series of the average solar magnetic field.

Given a complex network, a large variety of measures could be calculated in order to characterize its topological structure. In Ref. [34], the VG analysis is focused on the degree distribution. However, other measures may provide additional insight, or may turn out to be less useful, depending on the specific system studied. For instance, Muñoz et al. [27] have shown that some metrics are correlated with solar activity (degree centrality), or anti-correlated (eigenvector centrality), or remain constant throughout the variations in solar activity (clustering coefficient). This is a clear example that the complex network contains non-trivial information from a system, since metrics such as degree centrality are expected to be sensitive to variations due to their explicit dependence on the number of connections, while others, more elaborate metrics such as clustering coefficient, that quantify the grouping between neighbors, do not exhibit major variations throughout the cycle. All this suggests that the topology of the complex network contains non-trivial information about the physical state of the system, which is an important motivation for this work.

Thus, following Ref. [27], in this work, besides the degree distribution, we will focus on the clustering coefficient and various centrality measures, in order to examine the complex network from multiple perspectives. Furthermore, we will not only consider their average values, but also their distributions, by means of their respective critical exponents and Gini coefficients.

Thus, the interest of this project lies in characterizing nonlinear dynamical processes (in this case, the evolution of solar activity) through the complexity parameters that the system itself can provide, using a firmly tested statistical method. This paper is organized as follows. In Section 2, the dataset and analysis methods used to study them are described. Our results are provided in Section 3, and they are further summarized and discussed in Section 4.

## 2. Methodology

**Figure 1:**
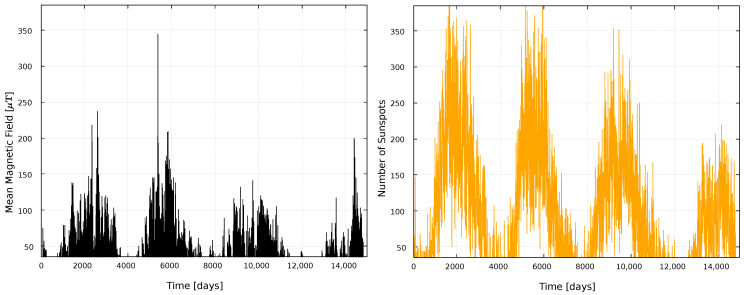
Time series used in this work. (**Left**) panel: mean magnetic field on the surface of the Sun. (**Right**) panel: number of sunspots.

To study solar activity, we use time series data of the number of sunspots observed on the solar surface [38], and Sun’s global magnetic field [39]. Both series will be considered between 1975 and 2015 with a one-day resolution, comprising Solar Cycles 21, 22, and 23, and the beginning of Solar Cycle 24. Data are shown in Figure 1. Three solar cycles are chosen, in order to have a relevant sample of solar activity in the last years. Since the WSO project started collecting data in 1975, while the sunspot data date back to 1818, we chose to collect the characteristic parameters of solar activity since 1975. We set as day zero the measurements of 16 May 1975, ending in day 14,185, corresponding to 16 December 2015.

Using these data, the method consists of constructing complex networks from the time series, using two variations of the visibility algorithms as connection criteria, aiming to extract statistical properties of the system. Thus, for each time series, two complex networks will be constructed, so that we will have a total of four networks.

### 2.1. Complex Networks

A complex network is a graph (set of nodes and their connections) with non-trivial statistical and topological properties. By representing an abstraction of a physical system, the definition of nodes and connections must consider both the properties of that system and the type of study to be performed. Therefore, different complex networks can be obtained from the same system, depending on the construction method.

After the networks are built, various measures must be calculated in order to study their possible correlation with solar activity along the 3 solar cycles previously mentioned. In particular, we considered node degree as a measure of connections, clustering coefficient, mean path length between nodes, and two centrality measures, namely, betweenness and eigenvector centrality.

### 2.2. Metrics

As mentioned in Section 2.1, we calculate several metrics to characterize the networks. Here we define the metrics that we will use in the rest of the paper, which are various way to the connectivity within the network. The node degree is the number of connections that a node has. We will use a normalized degree, to make it independent of the network size. If ν is an arbitrary node, and it has $n_{\nu }$ connections then its normalized degree is

(1) $$g\left(\nu \right)=\frac{n_{\nu }}{n}\;,$$

where *n* is the total number of nodes in the network.

The clustering coefficient of a node ν is the fraction of possible triangles which contain that node, and is defined as

(2) $$c\left(\nu \right)=\frac{1}{n}\frac{2T(\nu )}{g(\nu )(g(\nu )-1)}\;,$$

where T(ν) is the number of triangles containing node ν.

The mean path length corresponds to the average number of steps along the shortest paths for all possible pairs of network nodes. It then may be considered as a measure of the efficiency of information or mass transport on a network. It is defined as

(3) $$l=\sum_{s,t\in V}\frac{d(s,t)}{n(n-1)}\;,$$

where *V* is the set of nodes and d(s,t) is the minimum distance from node *s* to node *t*.

The centrality metrics measure the relevance of a node within the network. Betweenness centrality is the sum of the fraction of all-pairs shortest paths that pass through a node ν,

(4) $$b\left(\nu \right)=\frac{1}{n}\sum_{s,t\;\in \;V}\frac{\sigma (s,t|\nu )}{\sigma (s,t)}\;,$$

where *V* is the set of nodes, σ(s,t|ν) is the number of paths passing through some node ν other than s,t and σ(s,t) is the number of shortest paths between *s* and *t*. When s=t, then σ(s,t)=1 and if ν=s or ν=t, then σ(s,t|ν)=0 [40].

Eigenvector centrality computes the centrality for a node based on the centrality of its neighbors. The intuition is that a node is important, if it is connected to important nodes. The eigenvector centrality for a node ν is the ν-th element of the normalized vector x defined by

(5) Ax=λx,

where *A* is the adjacency matrix of the network with eigenvalue λ. The adjacency matrix is a square matrix of size *n*, where the element $A_{\mathrm{ij}}$ is 1 if there is a connection between vertex *i* and *j*, and 0 if there is no connection.

### 2.3. Connection Criteria

#### 2.3.1. Visibility Graph

We use the Visibility Graph (VG) algorithm, whose statistical properties have been studied in several publications [41,42]. The definition of VG for time series comes from the concept of visibility between nodes. Each element of the time series can be identified by a time *t* and its respective associated value x(t), which represents some physical quantity. Therefore, a node in the network is defined by the point (t,x(t)). Two nodes are connected if they “see” each other, i.e. if there is a straight line connecting them without being interrupted by other intermediate nodes. Formally, given a data series $X_{N}$, two arbitrary nodes $x_{a}$ and $x_{b}$ are connected if, for every node $x_{c}$ between them, then [42,43]

(6) $$x_{c}\leq x_{b}+\left(x_{a}-x_{b}\right)\frac{t_{b}-t_{c}}{t_{b}-t_{a}}\;.$$

#### 2.3.2. Horizontal Visibility Graph

A variant of VG known as Horizontal Visibility Graph (HVG) consists of restricting the visibility between nodes to a horizontal line. If ${\left\{X_{i}\right\}}_{\left\{i=1,2,\ldots ,N\right\}}$ is a time series of size *N*, two nodes *i* and *j* will be visible if for all nodes *n* such that i<n<j, then [41,44,45]

(7) $$X_{i}\;,\;X_{j}>X_{n}\;.$$

Therefore, horizontal visibility for two nodes occurs if there is no other node greater in magnitude between them.

## 3. Results

Construction of the complex network from the time series involves not only the decision on what will be regarded as a node, and what will be the criterion to connect two nodes, but also the length of the time window within which data will be considered. Figure 1 shows that solar activity has variations on various timescales. Thus, in order to obtain a better perspective of the solar activity, we carry out two analyses: a global analysis, considering the complete time series, and a local one, using time windows.

**Figure 2:**
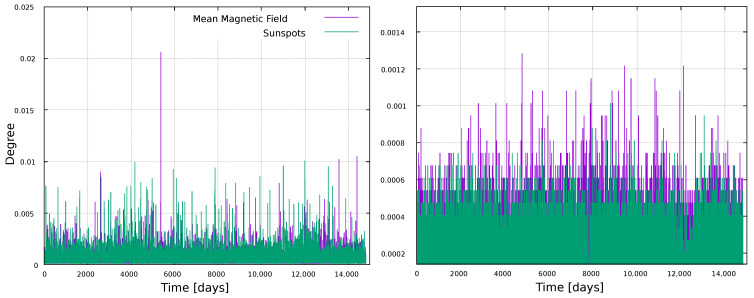
Degree for every node in the network, normalized by the size of the network, for the sunspots time series (green line) and the mean magnetic field time series (purple line). (**Left**) panel: VG; (**right**) panel: HVG.

We first consider the global analysis. Figure 2 shows the resulting degree for each node, normalized to the network size. Thus, the ordinate axis represents the fraction of nodes that each node is connected to. Results are shown in Figure 2.

We first observe that the number of connections is larger for the networks built by the VG method, which is expected, since the HVG method restricts visibility to a horizontal line, and therefore, less connections can be established. We also notice that there is no particular dependence of this metric with the solar cycles. There are some prominent values for the VG graph (time t∼5500), which match high values of the magnetic field (ascending phase of Solar Cycle 22, Figure 1), but as a general rule, no correlation is observed. Given the definition of the VG, one would expect that maxima in the time series would “see” more data in the rest of the time series, as they would tend to be unobstructed by intermediate points, thus leading to maximum degree. However, except for the very large maximum noticed above, this does not hold in general, due to the rapid fluctuations. This result suggests that the degree is too simple a metric to study these time series, thus justifying the need for more elaborate metrics.

The clustering coefficient measures the connectivity between nodes connected to a given node. It is related to the number of triangles formed by edges in the network, and thus it quantifies the degree to which nodes in a graph tend to cluster together. For the global network, one obtains average values equal to $C_{ss}\approx 0.721$ and $C_{mf}\approx 0.739$, for sunspots and magnetic field networks, respectively. These results indicate the presence of well-defined clusters within the time series, corresponding to solar cycles.

In the case of VG networks, the results obtained for both mean magnetic field and sunspots mean paths are $l_{mf}\approx 6.36$ and $l_{ss}\approx 5.73$, respectively. Both values are much smaller than the network size (*N* = 14,185), indicating that, although the network is large, nodes are close to one another on average, separated by at most 6 nodes.

Although these results are interesting, no particular dependence on the solar cycle is observed for the clustering coefficient and the shortest path length per node, for this global analysis, which is why we have not shown the corresponding plots.

**Figure 3:**
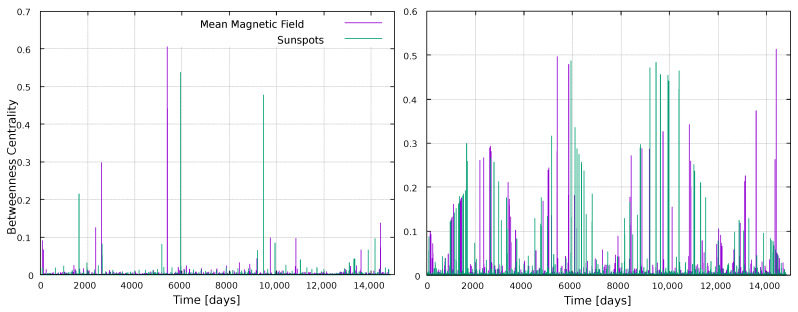
Betweenness Centrality for every node in the network, normalized by the size of the network. (**Left**) panel: VG method; (**right**) panel: HVG method.

The short distance between nodes notices above can be due, for instance, to a large number of connections between nodes, or to the presence of some highly important nodes, acting as bridges that connect different parts of the network. This can be quantified with the concept of betweenness centrality (BC) which, as other centrality measures, provide a way to assess the importance of nodes in the network. In this case, how important a node is to establish connection between nodes (see Figure 3).

The results obtained for this metric can be seen in Figure 3. In the case of VG results, we observe three zones where a few nodes have BC values much larger than other nodes in the network. For the HVG case, these zones are more distinguishable, as more nodes have large BC values. These results hold for both networks (sunspots and mean magnetic field). The most interesting feature of these results is the clear match between BC and variations in solar activity (Figure 1). As mentioned when Figure 2 was discussed, one would expect the highest points in the time series to be very well connected, as they should be able to “see” more nodes. However, this is not captured by the number of connections itself, as shown in Figure 2. This is unlike BC, where Figure 3 shows higher values of sunspots and mean magnetic field do not have, on average, more connections that the rest of the data, but they do play an important role in connecting nodes. In this sense, it is also interesting to note that the important nodes for the VG method are very few, where the nodes with large values of the BC belong to a narrow zone around solar maxima; whereas for the HVG, the BC has a wider distribution, following the sunspots and mean magnetic field time series in a smoother way.

**Figure 4:**
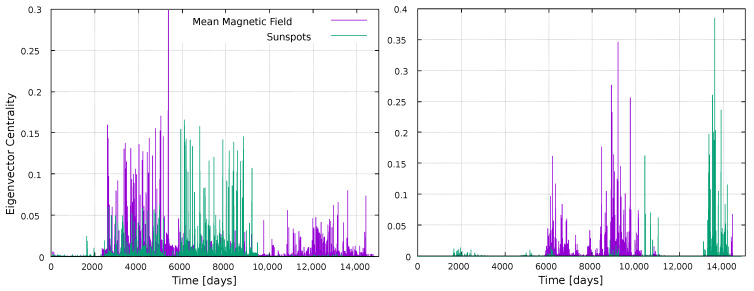
Eigenvector Centrality for every node in the network, normalized by the size of the network. (**Left**) panel: VG method; (**right**) panel: HVG method.

We have computed a further centrality measure, namely the Eigenvector Centrality (EC), based on the idea that a node is more important if it is connected to important nodes. The results are shown in Figure 4. Several features are interesting to observe, which differ from the previous plots. First, this metric clearly exhibits different results for each time series, as maximum values for the sunspots time series do not occur for the same nodes as for the magnetic field time series. This highlights the nontriviality and nonlinearity of the metrics, and supports the fact that it is interesting to calculate several metrics for a given network, as they may reveal different features. This is specially noticeable as both time series in Figure 1 show a similar behavior: sequences of maxima and minima which clearly mark all the solar cycles in the dataset, at essentially the same time. However, data are not the same, and results are clearly split by the eigenvalue centrality, not the metrics previously discussed.

Another interesting fact is that, in the VG case, maximum values of the eigenvector centrality tend to occur in between solar maxima, suggesting an anticorrelation with the solar cycle. Notice, for instance, the EC maxima for the magnetic field time series, between the 21st and 22nd solar cycle, and the maxima for the sunspots time series, between the 22nd and 23rd solar cycle. However, there are three intercycle time windows in the data set, but only two noticeable maxima of the EC for both the magnetic field and the sunspots time series. Since the EC is related to the importance of neighboring nodes, it is possible that the analysis is affected by boundary effects, as no data exist before and after the selected time window. However, it is worth noticing that several papers have been devoted to the prediction of features of the next solar cycle [46,47,48], such as its intensity. Since the EC for the VG seems to be most sensitive during the intercycle period, with different behaviors for each time series (e.g., the existence of maxima for the magnetic field series at day ∼13,000, while no important maxima occur for the sunspots series) it would be interesting to explore to what extent the EC could provide useful information on the next solar maximum before it is actually reached.

As for the HVG method, Figure 4 also shows that EC maxima do not occur simultaneously with sunspots maxima. Rather, they seem to cluster during the ascending or descending phase of cycles.

The analysis so far has focused on the value of network measures per node. However, the distribution of values may also have information, as it can provide insight about the physical processes underlying the network formation [49,50]. For instance, the HVG method typically leads to exponential degree distributions, P(k)∼exp(−γk), and it has been suggested that its decay exponent γ is related to the type of randomness [51]. Specifically, it has been suggested that a threshold value $\gamma_{\mathrm{un}}=\ln\left(3/2\right)\approx 0.405$ exists, such that $\gamma <\gamma_{\mathrm{un}}$ corresponds to a chaotic process, whereas $\gamma >\gamma_{\mathrm{un}}$ corresponds to a correlated stochastic process.

**Figure 5:**
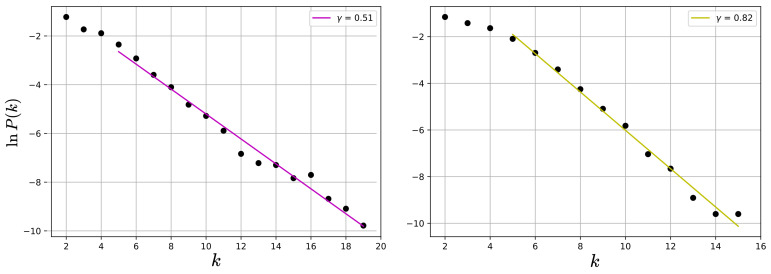
Semi-log plot of the degree distributions. (**Left**) panel: magnetic field time series; (**right**) panel: sunspots time series.

Thus, we calculate the probability distribution of a node having degree *k*, P(k), which corresponds to the fraction of nodes with *k* connections over the total amount of nodes. We can observe in Figure 5 that, indeed, networks for both time series follow an exponential distribution P(k). The value of γ is given by the slope of the linear fit of the semi-log distribution, and is computed considering the tail of the distribution [52], where a linear relation for lnP(k) and *k* holds. The estimated values of γ are 0.51 and 0.82 for the magnetic field and sunspots networks, respectively, which suggests an underlying correlated stochastic process [51].

Now, we employ moving time windows to follow the evolution of the network measures along the solar cycle. Two window sizes were chosen: 1-year windows, with a 1-month overlap; and 11-years windows, with a 1-year overlap. This leads to 493 windows of 1-year width, and 30 windows of 11-years width. We then plot results by associating, to each window, the time corresponding to its center.

**Figure 6:**
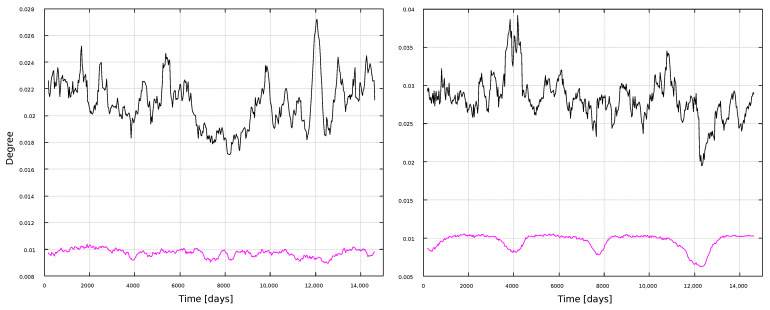
Degree for 1-year windows. (**Left**) panel: magnetic field networks; (**right**) panel: sunspots networks. Line color indicates the type of graph: VG (black line) and HVG (magenta line).

**Figure 7:**
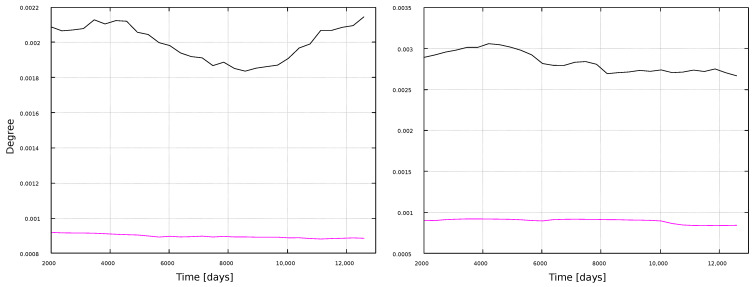
Degree for 11-year windows. (**Left**) panel: magnetic field networks; (**right**) panel: sunspots networks. Line color indicates the type of graph: VG (black line) and HVG (magenta line).

The same metrics as in the global analysis were calculated. Results for the degree are shown in Figure 6 and Figure 7. As expected, the VG method leads to larger number of connections than the HVG.

In general, results are consistent with the global results in Figure 2: the degree does not correlate with the solar cycle, regardless of the timescale of observation. The only exception is the HVG analysis for the sunspots time series, with 1-year windows (Figure 6), where clear minima close to solar minima can be found.

**Figure 8:**
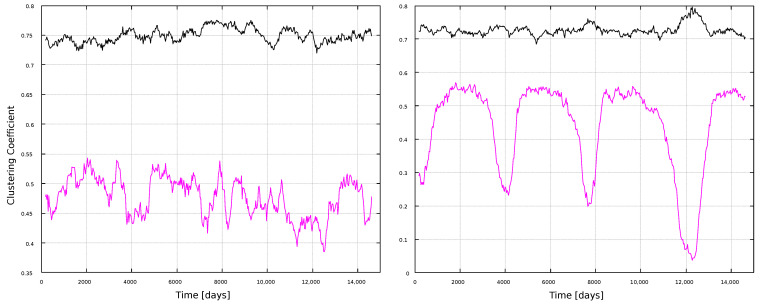
Clustering coefficient for 1-year windows. (**Left**) panel: magnetic field networks; (**right**) panel: sunspots networks. Line color indicates the type of graph: VG (black line) and HVG (magenta line).

**Figure 9:**
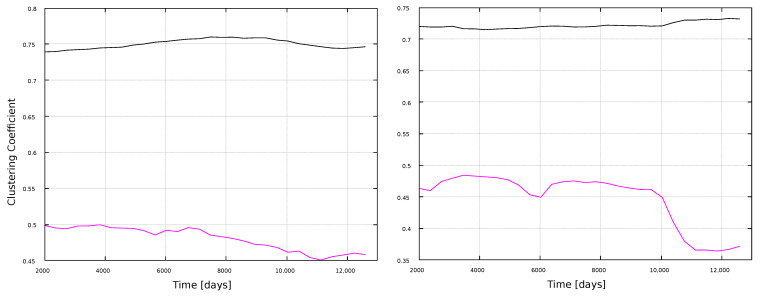
Clustering coefficient for 11-year windows. (**Left**) panel: magnetic field networks; (**right**) panel: sunspots networks. Line color indicates the type of graph: VG (black line) and HVG (magenta line).

Figure 8 and Figure 9 show the corresponding results for the clustering coefficient. Larger values are obtained for the VG method, for both window types. Furthermore, results do not show clear correlations with solar activity, but notably, the HVG method has the same kind of oscillating behavior as for the degree (Figure 6), but more pronounced (notice that both measures are normalized, so that their maximum possible value is 1). Interpretations of this behavior will be discussed later, in Section 4.

**Figure 10:**
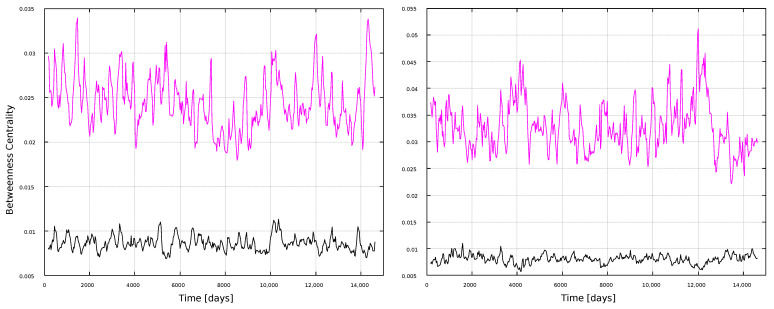
Betweenness centrality for 1-year windows. (**Left**) panel: magnetic field networks; (**right**) panel: sunspots networks. Line color indicates the type of graph: VG (black line) and HVG (magenta line).

**Figure 11:**
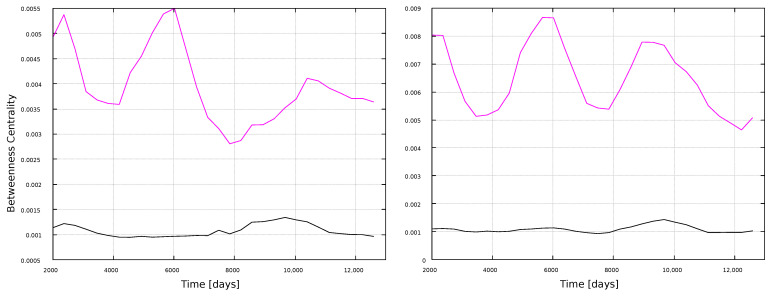
Betweenness centrality for 11-year windows. (**Left**) panel: magnetic field networks; (**right**) panel: sunspots networks. Line color indicates the type of graph: VG (black line) and HVG (magenta line).

Betweenness centrality results are shown in Figure 10 and Figure 11. We already noticed, in the global analysis, that BC was an interesting metric, due to its apparent sensitivity to the solar cycle (Figure 3). This is found here for the wider windows as well, Figure 11, showing peaks associated to maxima in solar activity (Figure 1). Thus, BC correlates well with solar activity, but if large timescales are studied (full time series in Figure 3, 11 years in Figure 11), and if the HVG is used. If shorter, 1-year windows are taken, or if the VG method is used, then the BC does not convey information on solar activity.

**Figure 12:**
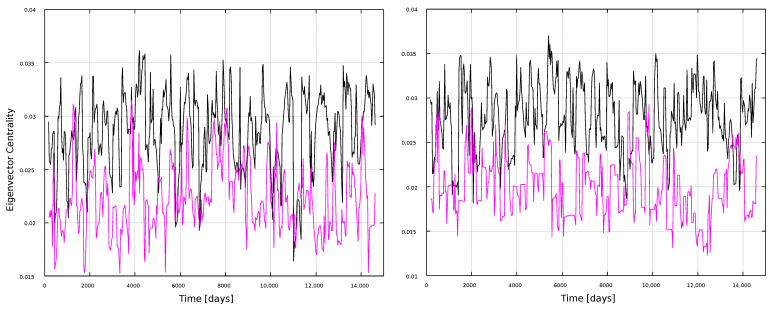
Eigenvector centrality for 1-year windows. (**Left**) panel: magnetic field networks; (**right**) panel: sunspots networks. Line color indicates the type of graph: VG (black line) and HVG (magenta line).

**Figure 13:**
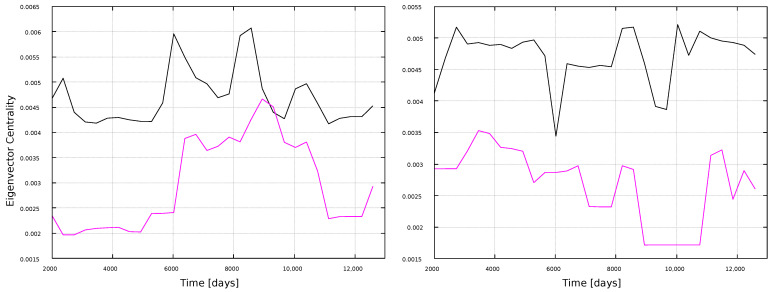
Eigenvector centrality for 11-year windows. (**Left**) panel: magnetic field networks; (**right**) panel: sunspots networks. Line color indicates the type of graph: VG (black line) and HVG (magenta line).

Finally, we compute the eigenvector centrality, shown in Figure 12 and Figure 13. Unlike Figure 4, this measure does not show interesting results for the local analysis, regardless of the moving window width, thus highlighting again the nontriviality of the results, as the usefulness of the network approach to follow solar activity depends both on the metric and the timescale observed.

**Figure 14:**
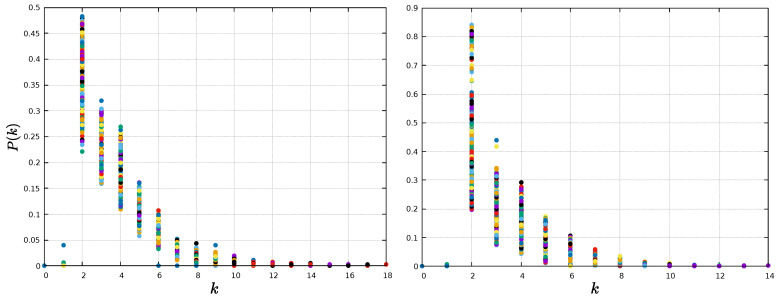
Degree distribution for each 1-year window. **Left** panel: magnetic field network; **right** panel: sunspots network. Each color represents a given window.

**Figure 15:**
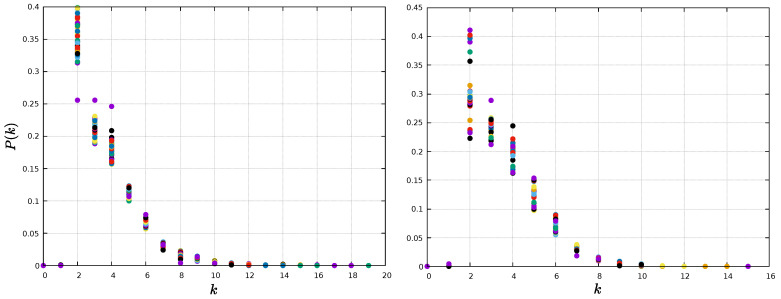
Degree distribution for each 11-year window. (**Left**) panel: magnetic field network; (**right**) panel: sunspots network. Each color represents a given window.

Regarding the degree distribution for the HVG method, all networks, for all time windows, exhibit an exponential topology, as in the case of the global networks, consistent with previous results for the HVG [51]. The degree distributions P(k) of every window are shown in Figure 14 and Figure 15, for 1-year and 11-year windows, respectively.

**Figure 16:**
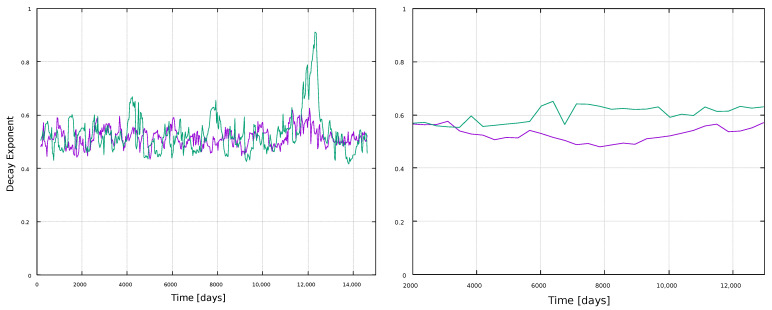
Evolution of the decay exponent for the degree distribution obtained from the HVG. (**Left**) panel: 1-year windows; (**right**) panel: 11-year windows. Line color indicate the time series used: magnetic field (purple line) and sunspots (green line).

The decay exponent γ for each window is shown in Figure 16. For both window types, 1-year and 11-year windows, similar values γ∼0.6 are found along the solar cycle. As mentioned before, this suggests correlated stochastic processes for every window, regardless of its length [51].

## 4. Discussion

In this work, we have studied and characterized solar activity using a complex network approach. By means of the visibility algorithms mentioned in Section 2, time series, and thus the Sun’s dynamics, are mapped into a complex network.

Various network metrics are calculated, which are related to node connectivity, edges density, distance between nodes and node relative importance. In general, larger values of the degree are found for the VG as compared with the HVG (Figure 2). This is a expected result since the HVG has a limited visibility, restricted to horizontal lines, and therefore less connections can be established.

For the global analysis, using the full time series, the most interesting metrics were the centrality measures. From Figure 3, we can observe sensitivity of the betweenness centrality to the solar cycle both for VG and HVG. This is a nontrivial result, because larger values of the time series would be expected to have more connections, because they should be more “visible” to other nodes. However, the degree itself does not capture variations in solar activity, whereas the betweenness centrality, which is a more elaborate measure, clearly does.

The eigenvector centrality also shows a dependence on the solar activity, but of a different kind. First, behavior is different for both time series (magnetic field and sunspots), thus this is the only metric, among those studied here, that distinguishes the physical quantity being observed. Besides, for the VG, maxima tend to lie close to solar minima, whereas for the HVG they tend to lie in the ascending or descending phases of the cycle. It is also interesting to observe the small values obtained for SC24, with the VG method, representing almost non-influence in the network, consistent with the substantially lower activity of this cycle with respect to other recent solar cycles. The HVG, on the other hand, yields different results to the VG ones. Considering that EC tends to show maxima outside solar maxima, and that it distinguishes between sunspots and magnetic field times series, it should be interesting to study to what extent this measure is able to provide information on the next solar maximum, before it is actually reached. We plan to examine this in more detail in the future.

The results for the local analysis are, in general, consistent with the global analysis. Figure 6 and Figure 7 show the expected result that the VG yields larger values for the degree than the HVG. It is also interesting to notice that the HVG degree shows a slight trend to decrease during its evolution, for the 1-year windows. However, one should take into account that values are normalized to the interval [0,1], and that the obtained values are very small ∼$10^{-2}$, thus the degree could be regarded as essentially constant, regardless of the size of the time windows. However, a similar and clearer trend is observed for the HVG, if other metrics are considered.

Figure 8 shows that, whereas the degree is different for VG and HVG, the clustering coefficient for the magnetic field and sunspots time series has about the same value, ∼0.75, for the VG method. On the other hand, the HVG method is able to pick variations associated to the solar cycle in the sunspots network. This sensitivity, though, is not present for the larger timescales, when the 11-year windows are used.

We have also observed interesting variations in the BC, for both the VG and HVG methods, with the larger scale time windows (11 years), as seen in Figure 11. This is consistent with the behavior found for the BC for the global analysis. One should consider, anyway, that calculated values are normalized to 1, and thus the variations shown in those figures are very small, of the order of $10^{-2}$. In this sense, the behavior of the BC for the global analysis is much stronger, but the subtle variations in the local analysis may also be interesting, specially because they are consistent with the local analysis for the degree and the clustering coefficients, which did not exhibit any special dependence on solar activity in the global analysis.

In general, most curves shown in Figure 6, Figure 7, Figure 8, Figure 9, Figure 10, Figure 11, Figure 12 and Figure 13 are featureless, with a few of them, as discussed above, showing noticeable variations which are consistent with the solar cycles. This is worth pointing out, because, although the sunspots and magnetic field time series clearly show variations in solar activity along solar cycles, and despite the interesting capabilities of the VG approach to identify statistical features in time series, it is interesting to point out when the VG can be most useful to study solar activity, and when it does not provide useful information.

The degree distributions are found to show an exponential behavior at the tail, as seen in Figure 5. The fast decay shows that on average, most nodes are connected to only a few nodes (degree probability is different from zero for k<6). However, the mean path length is very small compared with the size of the network, suggesting a small-world behavior. Basically, the information within magnetic field and sunspots networks is efficiently transferred toward the entirety of the system, locally and globally [53]. These results are preserved when the analysis is carried out in moving windows, as shown in Figure 14 and Figure 15. For this latter analysis, we also observe an essentially constant value of the decay exponent despite variations in solar activity, as shown in Figure 16.

Despite simple metrics like the degree may not exhibit strong dependence with solar activity, more elaborate ones like the clustering coefficient and centrality measures may show clear variations with the solar cycle. The centrality measures are particularly interesting, due to the strong dependence of the BC for the global analysis, and the distinction between the magnetic field and sunspots time series that the EC displays. Further analysis should be carried out to determine to what extent these findings may contribute to characterize future solar cycles in advance, but our findings highlight the nontriviality of the information extracted by each metric, as results depend on the algorithm used, and the time scale examined, complementing other, recent works, on complex network analyses for solar activity [54,55,56]. In particular, we have previously observed that observing with different network metrics the same time series (sunspots number), various results can be found, with some metrics correlated, others anti-correlated, and other being essentially constant along the solar cycle [27]. The present work also complements these results. Our findings also show that different time series, although they may be related to the same underlying physics (solar dynamics), are not equivalent for the VG algorithm, which is consistent with the fact that one cannot expect a single technique to provide all the possible information on a given phenomenon. Besides, the correlation of certain metrics, for some timescales, with solar activity, opens the question of to what extent this correlation may be used to either characterize solar cycles, or inform us about the dynamo process driving sunspots emergence and magnetic field variability along the solar cycle. We are currently working in some aspects of these questions, including the analysis of additional solar cycles, to understand in detail why some metrics perform better, and thus their connection to physical features, beyond the results presented here.

## Data Availability

Publicly available datasets were analyzed in this study.

## Abbreviations

The following abbreviations are used in this manuscript:

VG	Visibility Graph
HVG	Horizontal Visibility Graph
BC	Betweennes Centrality
EC	Eigenvector Centrality
